# Disenfranchised Guilt—Pet Owners’ Burden

**DOI:** 10.3390/ani12131690

**Published:** 2022-06-30

**Authors:** Lori R. Kogan, Cori Bussolari, Jennifer Currin-McCulloch, Wendy Packman, Phyllis Erdman

**Affiliations:** 1 Department of Clinical Sciences, Colorado State University, Fort Collins, CO 80523, USA; 2 Department of Counseling Psychology, University of San Francisco, San Francisco, CA 94117, USA; bussolari@usfca.edu; 3 School of Social Work, Colorado State University, Fort Collins, CO 80523, USA; jen.currin-mcculloch@colostate.edu; 4 Department of Psychology, Palo Alto University, Palo Alto, CA 94304, USA; wpackman@paloaltou.edu; 5 College of Education, Washington State University, Pullman, WA 99163, USA; perdman@wsu.edu

**Keywords:** guilt, parental guilt, dog, work–family conflict

## Abstract

**Simple Summary:**

Guilt is the unpleasant emotion associated with one’s behaviors, thoughts, or intentions, and it is based on the possibility that one may be in the wrong or others may have this perception. Parental guilt is one common type of guilt and is often associated with work–family conflict (WFC). WFC and related guilt have been found to be related to depression and anxiety. The current study was designed to explore dog owners’ guilt surrounding their dogs through an online anonymous survey. Results suggest that dog owners’ guilt and WFC associated with their dog are at similar levels to those reported in human family studies. Additionally, the relationship between dog owners’ guilt and discrepancy between their actual and ideal self, in regard to the role of a dog owner, also mirrored human-only family research. Because pet-related guilt is unrecognized, acknowledged, or supported, we suggest it is disenfranchised. In this period of post-COVID-19 pandemic time, as people return to work, it is important that companies and communities acknowledge pet owner guilt and pet-related WFC and help owners find practical, effective solutions.

**Abstract:**

Guilt refers to an unpleasant emotional state associated with one’s behaviors, thoughts, or intentions, and it is based on the possibility that one may be in the wrong or that others may have this perception. Parental guilt is one common subtype and is often associated with work–family conflict (WFC). WFC and related guilt have been found to be associated with depression and anxiety. Through an online anonymous survey, the current study was designed to explore dog owners’ guilt surrounding their dogs. Results suggest that dog owners’ guilt and WFC associated with their dog are at levels similar to those reported in human family studies. Additionally, the relationship between dog owners’ guilt and discrepancy between participants’ actual and ideal self, in regard to the role of a dog owner, also mirrored human-only family research. Because pet-related guilt is unrecognized, acknowledged, or supported, we suggest it is disenfranchised. As people return to work, in this period of post-COVID-19 pandemic time, it is paramount that companies and communities acknowledge pet owner guilt and WFC and help owners find practical, effective solutions.

## 1. Introduction

### 1.1. Guilt

Guilt refers to an unpleasant emotional state associated with one’s behaviors, thoughts, or intentions, and it is based on the possibility that one may be in the wrong or that others may have this perception [1,2]. It includes both affective and cognitive components, in which people believe that their behaviors have resulted in some form of negative outcome [3,4,5]. Guilt includes painful feelings, accompanied by a sense of responsibility and remorse, in response to specific circumstances. Guilty people blame themselves for something they may have or have not done [5]. In fact, an individual’s feelings of guilt may be independent of any actual physical, psychological, or emotional harm [6,7,8].

Guilt differs from shame. While guilt is characterized by internal, specific, and unstable attributions, shame is caused by internal, global, and stable attributions [9,10]. Guilt concerns a particular action, while shame pertains to the entire self [1]. Guilt can also be distinguished from fear of punishment; one can feel guilty even when there is no chance of punishment. Instead, the knowledge that one has harmed another being (whether this harm is real or imagined) can be enough to cause guilt [1]. Guilt has been linked with depression [11,12,13], anxiety [9,14,15], compassion fatigue, burnout, and decreased quality of life [6,16].

Parental guilt is one common subtype, and it is often associated with work and family conflict (WFC) [17,18,19,20]. Work and family conflict has two elements: family-to-work conflict (i.e., when family responsibilities negatively impact work) and work-to-family conflict (i.e., when work commitments negatively impact family) [19]. Within WFC, parental guilt is often described as a result of competing demands of work and family responsibilities [19,21,22]. This conflict occurs when the two roles are incompatible, resulting in an inability to meet the demands of either [23,24]. 

WFC and related guilt have been found to be associated with depression and anxiety [25,26], which can lead to negative behavior choices, including the decision to leave paid employment or feeling the need to take on too many responsibilities at home [27,28]. WFC and guilt can also have negative consequences for children whose parents attempt to cope with their guilt by becoming increasingly permissive [28,29].

Given the traditional gender role expectations in which mothers carry the primary responsibilities associated with caring for children, several studies suggest that WFC and related guilt appear to be more prevalent among women (both working and stay-at-home mothers), when compared to men [8,30,31,32,33,34,35]. Women who work report feeling guilty about not being home with their children [36,37], while stay-at-home mothers report feeling guilty for not earning money to better provide for their children [38]. Imposed by themselves and society at large, this sense of responsibility, combined with often unrealistically high standards for what it means to be a “good” mother, causes many women to feel inadequate and guilty [20,33]. Thus, although both fathers and mothers may experience conflict about competing demands of work and home, mothers may experience more personal distress and guilt when prioritizing work-related demands at the expense of home responsibilities, because these choices violate the traditional expectations of women and mothers [8,39,40,41].

### 1.2. Pets as Family

Research pertaining to WFC and parental guilt has centered around human family members, yet 70% of US homes have a pet [42], and the majority of pet owners (85% of dog owners and 76% of cat owners) consider their pets to be family members [43]. Pets have been shown to provide a plethora of benefits, including increased wellbeing [44,45,46,47,48,49,50], stress reduction [51,52,53], a buffer against depression [54,55,56,57,58] and anxiety [59,60,61], motivation to exercise [62,63,64], increased longevity [65,66], higher social functioning [67], enhanced social support [53,54], and a buffer against adverse family circumstances [68] and stressors associated with the COVID-19 pandemic [69,70,71,72,73,74]. It should be noted, however, that many studies have failed to find positive physical or psychological effects of animals [75,76,77].

Regardless of how people define family most agree that their family is a critical component in their lives. As noted by Bowen [78] and others [43,44,54,79,80,81], pets are often an integral part of the family structure. This exemplifies the expanding fluidity of the word “family”, moving beyond the traditional definition as a group of two or more persons related by birth, marriage, or adoption who live together [82]. A more expansive definition of family can be of benefit; people who include a wider array of entity types in their personal definition of family appear to experience better mental and physical health [83]. In particular, many people view their pets as children [84,85,86,87] and think of themselves as ‘pet parents’. A parent can be defined as a person who brings up and cares for another [88], i.e., someone responsible for the physical, psychological, and financial support for those they care for [85]. Both men and women can view their pets as children; it appears that the main difference is the acceptance of the term “parent” to define their role; women appear to feel more comfortable using the term than men [89].

### 1.3. Pet Owners and Guilt

Even though many people view their pets as family members, and even children, parental guilt regarding pets has not been studied. To our knowledge, the only research pertaining to guilt and pets revolves around relinquishment [90,91,92], medical, and, especially, end-of-life care and euthanasia-related decisions [93,94,95,96,97]. The current study was designed to test the following hypotheses regarding dog owners and guilt:

1. Since pets are often viewed as family members, we hypothesize that pet owners’ guilt, as associated with self-discrepancies and WFC, will have similarities to that reported in studies examining parental guilt and WFC related to human children/family.
2. The discrepancy between owners’ views of themselves and the ideal dog owner will be a significant predictor for owner guilt, as measured by the Guilt about Parenting Scale-Dogs (GAPS-D).
3. Owners use a variety of compensatory behaviors/thoughts to cope with their guilt and are more likely to use compensatory behaviors/thoughts as guilt increases.

## 2. Materials and Methods

### 2.1. Sample

Survey respondents were recruited 15 April–17 April 2022, through Prolific, an open online marketplace providing access to potential survey respondents, in which respondents receive small monetary compensation for completing surveys (participants received $1.55 for completing this survey). Diversity of participants recruited through platforms such as Prolific is higher than typical Internet samples or North American college-based samples, and the quality of data collected met or exceeded the psychometric standards considered acceptable in published research in the social sciences [98]. In order to minimize the influence of geographic and cultural differences on respondent data, the survey was made available only to adult dog owners (18 years or older) who reside in the United States, were the primary caretakers of their dog, and had owned the dog for at least six months.

### 2.2. Instrument

An online, anonymous, cross-sectional survey was developed using Qualtrics (Qualtrics, Inc., Provo, UT, USA). The study was approved by the Colorado State University Institutional Review Board (IRB #3454). The survey began with an introduction that explained the purpose of the study and instructions regarding how to answer the survey if they have more than one dog. They were instructed that, if you had more than one dog, please answer the following survey questions for the dog whose name begins with the letter that comes first in the alphabet. For example, if you have a dog named Fluffy and another dog named Roy, please answer the survey about Fluffy, since “F” comes before “R” in the alphabet.

#### 2.2.1. Discrepancy between Ideal versus Actual Dog Owner Descriptors

The first part of the survey consisted of two series of 10 adjectives that can be used to describe dog owners (e.g., caring, forgiving, and nurturing). A list of 17 adjectives were selected by the researchers and piloted by dog owners to determine the final 10 adjectives selected most frequently to define an ideal dog owner. Participants were first asked to indicate how well each adjective describes themselves as a dog owner using a 5-point Likert scale, with 1 = not at all characteristic and 5 = very characteristic. They were then given the same list of adjectives and asked to indicate how well each adjective describes an ideal dog owner using the same rating scale. Adjective checklists have been found to be valid assessments of self-discrepancies in previous research [31,99]. An average was calculated across all 10 adjectives for both the participant (i.e., actual) and ideal dog owner. Discrepancy scores were calculated by subtracting the ‘‘actual’’ average from the ‘‘ideal’’ average. Higher scores indicate a greater level of discrepancy between how dog owners perceive themselves and the ideal dog owner.

#### 2.2.2. Dog Bond

Participants’ bond level with their dog was assessed through the use of three questions, which are included in the CENSHARE Pet Attachment Scale [68,100] These questions included: (1) “How often do you spend time each day playing with or exercising your pet?”, (2) “When you feel bad, how often do you seek your pet for comfort?”, and (3) “How often do you consider your pet to be a member of your family?”. They were answered using a four-point Likert scale, with options including “never”, “sometimes”, “often”, or “almost always”. The responses were summed to give a total bond score from 3 (weak) to 12 (strong).

#### 2.2.3. Guilt about Dog Parenting Scale (GAPS-D)

Modeled after the Guilt about Parenting Scale developed by Haslam et al. [101], this scale consisted of 10 items and was scored using a 7-point Likert scale, with 1 = “definitely do not agree” and 7 = “definitely agree”. The questions in the scale were the same as those used by Haslam [101], with the only difference being the substitution for the word ‘child’ with the word ‘dog’. Scores from the scale can range from 10 to 70. Higher scores indicate a greater sense of guilt related to dog ‘parenting’. The scale has been shown to correlate with PANAS-X’s [102] sub-dimension of general guilt (r = 0.64, *p* < 0.001). The Cronbach alpha for this scale has been reported to be 0.89 [101].

#### 2.2.4. Dog Owner Guilt

Participants were next asked to indicate their agreement level to twenty-one statements pertaining to areas of possible guilt using a 5-point Likert scale, with 1 = “strongly disagree” to 5 “strongly agree” (or NA). Examples of these questions include: “I feel guilty I do not walk my dog (at all or enough)”, “I feel guilty I do not pet my dog enough”, and “I feel guilty when I board my dog”.

#### 2.2.5. Work Dog Conflict (WDC)

The survey then included a series of 5 questions modeled after the work–family conflict (WFC) scale created by Netemeyer [103]. The questions remained the same, with the only difference being the substitution for the word ‘family’ with the word ‘dog’. Examples of questions include “The demands of my work interfere with time spent with my dog” and “The amount of time my job takes up makes it difficult to fulfill my dog-related responsibilities”. Participants were asked to indicate their agreement to each statement using a 5-point Likert scale with 1—“strongly disagree “ and 5—“strongly agree”. Cronbach’s Alpha for the WFC has been reported to be 0.88 [103].

#### 2.2.6. Compensatory Behaviors, Feelings, and Thoughts

A series of items were included in the survey to assess compensatory behaviors, feelings, and thoughts as a result of dog-related guilt. These included four statements, indicating a reduction in socialization/recreation (e.g., “Say no to evening social events because you feel guilty leaving your dog at home”), coping thoughts or feelings (e.g., “Remind yourself that your dog has a good life when you feel guilty” and “Feel resigned to feeling guilty about your dog”), and taking their dog with them (“Bring your dog to events and/or work because you feel guilty leaving your dog at home”). 

#### 2.2.7. Demographics

At the end of the survey, participants were asked to answer a series of demographic questions that included: age, ethnicity, race, marital status, education, gender, and children under the age of 18 living in the house. 

### 2.3. Statistical Analysis

Statistical analyses, including descriptive statistics, factor analysis, and linear regression, were conducted with IBM SPSS Version 26 (IBM, Armonk, NY, USA). Significance level (α) was *p* = 0.05, and all tests were two-tailed. Descriptive statistics were calculated to characterize owner demographics. Factor analysis was used to create guilt factors from a series of 21 statements pertaining to dog owner guilt. Kruskal–Wallis (KW) nonparametric analyses of variance tests were used to explore the relationship between GAPS scores and owner demographics, including marital status (single, partnered/married, divorced/widowed/other), education (less than high school/high school, 2 year college, 4 year college, graduate/professional school), age (under 30 years of age, 30–39, 40–49, 50 years or older), gender (male, female), and children under 18 in the home (yes, no). We next performed a multiple linear regression analysis using discrepancy between ideal versus actual and five guilt factors (time/attention, alone, away from home, physical health, furniture), while controlling for owner gender. Results of exploratory KW analyses were used to guide the selection of predictors for the multiple regression model.

## 3. Results

A total of 592 surveys were completed by adult dog owners residing in the United States who had their dog for at least six months. Eight participants were eliminated because they did not complete the attention questions correctly or did not have a dog for at least six months. The respondents were predominantly under the age of 40 (58%), White (84%), and non-Hispanic/Latinx (92%) and included 283 (48%) females and 289 (49%) males, 15 (3%) nonbinary, and five (1%) who chose to not answer or selected ‘other’. Most participants had a four-year college degree or higher (339; 57%) and reported their relationship status as partnered/married (304; 51%). The number of respondents who reported having children in the home under 18 years of age was 159 (27%) (Table 1).

### 3.1. Discrepancy between Ideal versus Actual Dog Owner Descriptors

Participants rated how well the 10 adjectives described themselves as a dog owner. The mean scores for the 10 adjectives were calculated to create a total score for actual dog owner perception (M = 4.46; SD = 0.49, range = 2.80–5.00; α = 0.894). Similarly, the mean scores for the 10 adjectives were calculated to create a total score for the perception of an ideal dog owner (M = 4.77; SD = 0.36, range = 2.60–5.00; α = 0.895). The mean actual dog owner score was subtracted from the mean ideal dog owner score to create a new variable, i.e., the discrepancy between participants’ perceived self and an ideal dog owner. Participants between 30–39 years of age reported higher discrepancy than other ages (F (4) = 3.45, *p* = 0.008). No differences were found based on gender, education, marital/relationship status, or children. 

### 3.2. Dog Bond

Participants’ bond level with their dog was assessed through the use of three questions. The responses were summed to give a score for dog attachment from 3 (weak) to 12 (strong). Responses ranged from 4 to 12, with a mean of 9.84 (SD = 1.57) (α = 0.548). Females reported a higher bond scale than males (F (1) = 9.39, *p* = 0.002). There were no differences based on age, education, marital status, or children. 

### 3.3. Guilt about Dog Parenting Scale

The guilt about dog parenting scale’s (GAPS-D) Cronbach alpha was 0.89. Scores ranged from 10 to 70 (mean = 44.75, SD 12.35). Females had higher GAPS-D scores than males (F (1) = 11.76, *p* < 0.001). Participants 60 and older had lower GAPS-D scores than participants under 40 years of age (F (4) = 3.44, *p* = 0.009). No differences based on education, marital status, or children were found. 

### 3.4. Dog Owner Guilt

Factor analysis was conducted on 21 statements pertaining to possible areas of dog-related guilt. After assessing for missing data and collinearity between the variables, inter-correlation among items was assessed using a correlation matrix, in order to ensure all items were contributing to the latent construct. Since no items correlated at a level higher than 0.8, each item was determined to be conceptually distinct, with no multicollinearity or singularity issues. Next, the 21 items were examined using the Kaiser–Meyer–Olkin measure of sampling adequacy and deemed acceptable at 0.88. Bartlett’s test of sphericity was significant (χ^2^ (136) = 4667.57, *p* < 0.001), and the communalities were all above 0.30. Therefore, the 21 items were found to share some common variance with other items and deemed to be suitable for factor analysis. 

Principal axis factor, with principal component analysis as the extraction method and direct oblimin with Kaiser normalization as the rotation method, was used. Direct oblimin was used after determining that the component correlation values exceeded the acceptable value of 0.32. The results confirmed the existence of five factors, which explained 68.67% of the variance and was preferred because of the ‘leveling off’ of eigen values on the scree plot after the five factors. Five statements were excluded due to low factor loading. These included: ‘I feel guilty when sometimes I think my dog is a burden’, ‘I feel guilty when I can’t afford higher priced food’, ‘I feel guilty when I do not take my dog to doggie day care (at all or enough)’, ‘I feel guilty when I do not take my dog to the vet for preventative care more often’, and ‘I feel guilty when I do not train my dog (at all or enough)’. 

The five guilt factors include: time/attention (α = 0.834), away from home (α = 0.764), leave alone (α = 0.826), physical health (α = 0.707), and furniture rules (α = 0.920) (Table 2 and Table 3), explaining 38.25%, 10.33%, 7.81%, 6.37%, and 5.91% of the variance, respectively.

### 3.5. Work–Family Conflict—Dog (WFC-D)

Because some people might not have been working outside the home, a NA option was added to the WFC-D scale. Excluding those who selected NA left 494 responses. The range of scores for the sum of the five WFC-D scale items was 5–25 (M = 13.34, SD = 5.50) (α = 0.935). The mean score was 2.67 (SD = 1.10). A total of 30.6% (*n* = 151) of people scored above the cutoff of the WFC scale of 16.69 [103], indicating they struggle with WFC dog-related conflict. Males reported higher WFC-D than females (F(1) = 8.80, *p* = 0.003). Participants younger than 50 years of age reported higher WFC-D than those 50 years of age or older (F(4) = 5.04, *p* < 0.001). Pearson correlation between WFC-D and GAPS-D was 0.414 (*p* < 0.001), indicating a significant correlation between dog owner guilt and work–family dog-related conflict. No differences based on education, marital status, or children were found.

### 3.6. Multiple Linear Regression

To determine the variables to include in the multiple linear regression, Kruskal–Wallis tests were performed to explore the relationship between GAPS-D scores and owner demographics (Table 4). With the exception of gender, no owner demographics were significantly associated with GAPS-D scores and, therefore, were not entered into the regression model. We performed a multiple linear regression analysis to predict GAPS-D using WFC-D, discrepancy between ideal versus actual dog owner descriptors, dog bond, and the five dog-related guilt factors (time/attention, leave alone, away from home, physical health, and furniture), controlling for owner gender. All variables were entered simultaneously. The multiple regression model (Table S1, supplementary material) predicting the GAPS-D score was significant (F(9) = 58.81, *p* < 0.001), with an R^2^ of 0.522. Significant predictors of GAPS-D score included four of the guilt factors (time attention, B = 0.076; *p* < 0.001; away from home, B = 0.034; *p* = 0.008; leave alone B = 0.097; *p* < 0.001; physical health B = 0.034; *p* = 0.018), owner gender (females had higher GAPS-D scores; B = 0.205; *p* = 0.012), discrepancy between ideal versus actual dog owner descriptors (B = 0.352; *p* = 0.001), and WFC-D (B = 0.253; *p* < 0.001). The largest predictors of GAPS-D score were WFC-D, discrepancy between ideal versus actual dog owner descriptors, and owner gender.

### 3.7. Compensatory Behaviors, Feelings, and Thoughts

A series of items were included in the survey to assess the potential compensatory behaviors/feelings participants might engage in as a way to cope with their dog-related guilt. These included three statements reflecting a reduction in socialization/recreation (e.g., “Say no to evening social events because you feel guilty leaving your dog at home”); coping thoughts or feelings (e.g., “Remind yourself that your dog has a good life when you feel guilty” and “Feel resigned to feeling guilty about your dog”); and behaviors (e.g., “Bring your dog to events and/or work because you feel guilty leaving your dog at home”, “Spend time with my dog at the expense of other family members because I feel guilty”). Approximately 40% of respondents indicated reducing their socialization/recreation time in response to their dog-related guilt. Nearly 35% reported spending time with their dog at the expense of other family members. Over 75% of respondents reported reminding themselves that their dog has a good life when they feel guilty, yet 42% reported, at least sometimes, feeling resigned to feeling guilty about their dog (Table S2, supplementary material). Kruskal–Wallis tests were performed to explore the relationship between GAPS-D scores and compensatory behaviors, feelings, and thoughts. All compensatory behaviors, feelings, and thoughts were significantly associated with GAPS-D scores, whereby higher GAPS-D scores were associated with an increased likelihood of utilizing compensatory mechanisms (Table S3, supplementary material).

## 4. Discussion

This paper explored pet owners’ guilt (measured by the GAPS-D) and work–family conflict (measured by the WFC-D) and found guilt and conflict levels similar to those reported in studies examining these issues in human family studies. Additionally, our results pertaining to the relationship between pet owners’ guilt and discrepancy between participants’ actual and ideal self, in regard to the role of a dog owner, also mirrored human-only family research. 

To assess pet owner guilt, we modified the GAPS assessment tool, a 10-item unidimensional measure capturing the cognitive-emotional features of parenting guilt, by substituting the word ‘dog for ‘child’. Participants’ guilt scores (mean = 44.75) were comparable to those reported in studies assessing parents and their human children. Haslam et. al. [101] reported a mean of 49.17. Chakraborty [18] reported means, prior to intervention, of 48.27 and 46.29. Females in our study had higher GAPS-D scores than males (F (1) = 11.76, *p* < 0.001). Studies with human family members have also reported higher parental guilt for women, when compared to men [28,104,105]. We also found that participants 60 years of age and older had lower GAPS-D scores than participants under 40 years of age (F (4) = 3.44, *p* = 0.009). The reason for this difference is not known, but we suggest this might be due to the fact that participants over the age of 60 might have fewer responsibilities outside the home than younger dog owners. It is also possible that older participants have had more experience caring for dogs and cope better with feelings of guilt.

We measured dog-related work–family conflict by revising the WFC [103] to reflect conflict pertaining to the participants’ dogs. We found that approximately one-third of owners scored above the conflict cutoff score [103], indicating that they struggle with dog-related WFC conflict. Similar means have been reported in previous studies [106,107] exploring WFC with human family members. Participants younger than 50 years of age in our study reported higher WFC-D than those 50 years of age or older, perhaps due to time allocated to work. We did not ask participants how many hours a week they worked. It is possible that those over 50 years of age work fewer hours outside the home and, therefore, experience less dog-related WFC. Males in our study reported higher WFC-D than females. While traditionally, WFC has been viewed as more prevalent with women, more recent studies have suggested that men and women report similar levels of WFC [108,109,110,111]. When assessing the relationship between GAPS-D and WFC-D, our results (r = 0.414) mirror that reported by Haslam et al. [101] (r = 0.42) between GAPS scores and work–family conflict (as measured with the WAFCS). We also found WFC-D to be a significant predictor of GAPS-D scores. 

Similarities between the parenting of human children and dogs extended to our assessment of the discrepancy between owners’ actual and ideal self, in terms of how they view the role of dog owner. The self-discrepancy theory [112] suggests that the degree of discrepancy between the actual and ideal self is associated with guilt and shame [99,113,114]. Studies have shown that when women experience discrepancy between their actual sense of self, in relation to being a mother, and their ideal sense of a mother, they feel maternal guilt and shame [31,34,37]. Our results suggest a similar process, whereby discrepancy, in this context between an actual and ideal dog owner, predicts dog owner guilt. 

In addition to discrepancy between ideal and actual perceptions of dog ownership and dog-related WFC, we found that four out of the five newly developed dog-related guilt factors were significant predictors of GAPS-D scores. These factors included guilt related to time/attention, being away from home, leaving their dog alone, and the dog’s physical health. The largest predictors of these factors were guilt pertaining to time/attention and leaving their dog alone. Lastly, we found that owner gender was also a significant predictor of dog-related guilt measured by the GAPS-D. Numerous studies have found that mothers have higher levels of parental guilt, when compared to fathers [8,104,115,116]. 

Together, our findings suggest that dog owners’ experiences of dog-related guilt and WFC mirror that reported by parents, in regard to their human children, in both prevalence and severity. Given the pervasiveness of guilt, we might ask if it serves any purpose. It has been suggested that guilt is correlated with empathy [117], whereby empathic people are more likely to experience guilt than those who are less empathic [99], and can strengthen social attachments. Guilt can motivate relationship-enhancing behaviors, thus helping to enforce social norms, such as concern, respect, and positive treatment [1]. Guilt can make people less likely to hurt or disappoint others; when they do, guilt can make them attempt to change their behaviors. This impact of guilt can be seen in our study results, in which higher levels of guilt were associated with a variety of compensatory behaviors, including owners’ decisions to reduce the time they spend away from home and amount of time they leave their dog alone. Another way in which participants attempted to reduce their guilt was to remind themselves that their dog had a good life, a coping mechanism endorsed by over 75% of respondents. 

Additionally, guilt can be a way in which less powerful beings get their way. Because guilt does not depend on formal power or influence, and may even work best in the absence of power, it can be an effective tool in restoring equity. Guilt in this context, however, only works if the guilty person cares about the other being. Dogs’ abilities to ‘make’ their owners feel guilty is a prevalent theme in dog owner conversations and social media postings. It is possible that the combination of owners’ tendency to anthropomorphize and the paedomorphic (childlike) facial characteristics [118] in many breeds trigger owners’ feelings of guilt. Paedomorphic facial features, especially those in which the upper facial muscle contractions lift the brow to increase the perceived height and size of the orbital cavity (i.e., puppy eyes), may exacerbate this effect. Large eyes, relative to the rest of the face, are a prominent feature in human infants and associated with perceived cuteness and a motivation to nurture [119,120]. These infantile facial features are similarly preferred in dogs and have been shown to increase their perceived cuteness [121]. Yet, despite the potential positive aspects of guilt on social relationships, as noted above, numerous studies have found that guilt is linked to anxiety [9,30], depression [122,123], and psychopathology [124,125].

Regardless of the similarities found in this study between dog-related guilt and WFC and parental guilt and WFC for human children, to our knowledge, the only published dog-related guilt research is limited to health issues (in particular, end-of-life) and relinquishment. Guilt related to being a dog owner is unrecognized and unacknowledged. For this reason, we suggest that pet owner guilt is disenfranchised. 

## 5. Conclusions

Traditionally, the word disenfranchised has been linked with grief to describe the pet bereavement experience [126]. Disenfranchised grief is the failure to understand the meaning and experience of another, thereby invalidating the bereaved person’s loss [127]. Doka [128] defined disenfranchised grief as the grief that people experience when they incur a loss that is not acknowledged or supported. Disenfranchised grief can complicate the bereavement process and deepen or prolong negative emotional reactions [129,130,131,132]. Death of a companion animal, for many, is a disenfranchised grief, resulting in a sense of isolation and separation from important social relationships [133,134,135,136]. 

Because pet-related guilt is similarly unrecognized, acknowledged, or supported, we suggest it is disenfranchised. Yet, similar to parental guilt [30,123,124], it may lead to feelings of anxiety, depression and poor psychological health. 

Furthermore, we suggest that this time period, when people are returning to work after the isolation and restrictions necessitated by COVID-19, is a critically important time to recognize the potential negative impacts of pet-related guilt. This is especially true for owners’ guilt related to being away from home and leaving one’s pet alone. Studies conducted in the early stages of the COVID-19 pandemic found that many pet owners, while enjoying the additional time they were able to spend with their pets, were concerned how their pets would handle the transition when they had to return to work outside the home [70,71,72]. 

As people return to work [137], many struggle with pets who have grown accustomed to their owners being home all day. It is paramount that companies and communities acknowledge pet owner guilt and WFC and help owners find practical, effective solutions. Examples include increased schedule flexibility, psychoeducation about the detrimental aspects of guilt, and validation of the owners’ guilt experiences through support groups and individual therapy. Only through legitimizing and validating the feelings of guilt and conflict felt by many pet owners can we hope to ease their transition back to work and ensure families stay intact. 

## Figures and Tables

**Table 1:** Participant demographics.

Category	N	(%)
Age		
Under 30 years	191	32.3
30–39	152	25.7
40–49	93	15.7
50–59	90	15.2
60–69	50	8.4
70 years or older	15	2.5
Prefer to not say	1	0.2
Race		
African American/Black	21	3.5
Asian	33	5.6
Biracial/multiracial	23	3.9
Middle Eastern	2	0.3
Native American/Indigenous	1	0.2
Native Hawaiian/Pacific Islander	2	0.3
White/Caucasian	497	84.0
Other	9	3.5
Prefer to not say	4	0.7
Ethnicity		
Hispanic/Latinx	44	7.4
Not Hispanic/Latinx	542	91.6
Prefer to not say	6	1.0
Gender		
Male	289	48.8
Female	283	47.8
Non-binary	15	2.5
Other	2	0.3
Prefer to not say	3	0.5
Education		
Less than high school	6	1.0
High school	151	25.5
Vocational/trade school/2-year college degree	94	15.9
College (4-year degree)	248	41.9
Graduate school/professional school	91	15.4
Prefer to not say	2	0.3
Marital/relationship status		
Single	227	38.3
Partnered/married	304	51.4
Divorced	47	7.9
Widowed	3	0.5
Prefer to not say	11	1.9
Children		
Yes	159	26.9
No	430	72.6
Prefer to not say	3	0.5

**Table 2:** Rotated component matrix for five dog-related guilt factors.

	Time/Attention	Away from Home	Leave Alone	Physical Health	Furniture Rules
I feel guilty when I do not play with my dog more/enough	0.819				
I feel guilty when I do not pet my dog enough	0.772				
I feel guilty when I do not walk my dog (at all or enough)	0.714				
I do not groom my dog (at all or enough)	0.623				
I feel guilty when I am too tired to pay attention to my dog	0.570				
I feel guilty when I work long shifts away from home		0.763			
I feel guilty when I board my dog		0.706			
I feel guilty when I go to work		0.649			
I feel guilty when I go out of town/travel and leave my dog at home		0.625			
I feel guilty when I leave the house for short periods of time (less than 2 h) and leave my dog at home			0.842		
I feel guilty when I leave the house for long periods of time (more than 2 h) and leave my dog at home			0.768		
I feel guilty when I leave my dog alone			0.688		
I feel guilty when I do not brush my dog’s teeth (at all or enough)				0.832	
I feel guilty when I do not regularly get my dog’s teeth cleaned at the veterinarian				0.731	
I feel guilty when I do not run with my dog (at all or enough)				0.620	
I feel guilty when I do not allow my dog on the furniture (at all or enough)					0.913
I feel guilty when I do not allow my dog on my bed (at all or enough)					0.910

**Table 3:** Factor analysis—five dog-related guilt factors.

Time/attention α = 0.834
I feel guilty when I do not play with my dog more/enough
I feel guilty when I do not pet my dog enough
I feel guilty when I do not walk my dog (at all or enough)
I do not groom my dog (at all or enough)
I feel guilty when I am too tired to pay attention to my dog
Away from home α = 0.764
I feel guilty when I work long shifts away from home
I feel guilty when I board my dog
I feel guilty when I go to work
I feel guilty when I go out of town/travel and leave my dog at home
Leave alone α = 0.826
I feel guilty when I leave the house for short periods of time (less than 2 h) and leave my dog at home
I feel guilty when I leave the house for long periods of time (more than 2 h) and leave my dog at home
I feel guilty when I leave my dog alone
Physical health α = 0.707
I feel guilty when I do not brush my dog’s teeth (at all or enough)
I feel guilty when I do not regularly get my dog’s teeth cleaned at the veterinarian
I feel guilty when I do not run with my dog (at all or enough)
Furniture rules α = 0.920
I feel guilty when I do not allow my dog on the furniture (at all or enough)
I feel guilty when I do not allow my dog on my bed (at all or enough)

**Table 4:** Kruskal–Wallis test results assessing the association between GAPS-D scores and owner demographics (*n* = 494).

	H (df)	*p*
Age	5.63 (3)	=0.131
Education	5.85 (3)	=0.119
Children	1.24 (1)	=0.266
Marital/relationship status	3.13 (2)	=0.209
Gender	4.76 (1)	=0.029

## Data Availability

Data available upon request.

## Supplementary Materials

The following supporting information can be downloaded at: https://www.mdpi.com/article/10.3390/ani12131690/s1, Table S1: Results of the Multiple Linear Regression Model Predicting GAPS-D Score as a Function of Dog Bond, Owner Gender, WFC-D, Dog-Related Guilt Factors, and Discrepancy between Ideal versus Actual Dog Owner Descriptors; Table S2: Reported Compensatory Behaviors, Feelings, and Thoughts as a Result of Dog-Related Guilt, Table S3: Kruskal-Wallis Test Results Assessing the Association between GAPS-D Scores and Compensatory Behaviors, Feelings and Thoughts.
