# Supplementary material for: Disenfranchised Guilt—Pet Owners’ Burden
Source: Animals (Basel). 2022 Jun 30;12(13):1690. doi: 10.3390/ani12131690 (PMC9264879; doi:10.3390/ani12131690)
Supplement: Supplementary file 1 [file animals-12-01690-s001.zip › animals-1767059-supplementary.pdf]

Supplementary material

Table S1. Results of the Multiple Linear Regression Model Predicting GAPS-D Score as a Function of Dog Bond, Owner Gender, WFC-D, Dog-Related Guilt Factors, and Discrepancy between Ideal versus Actual Dog Owner Descriptors

ANOVA

Model	Sum of Squares	df	Mean Squares	F	Sig.
Regression	387.40	9	43.04		
Residual	355.01	485	0.73	58.81	<0.001
Total	742.40	494			

Coefficients* (Dependent Variable: GAPS score)

Variable	Coefficient (B)	Std. Error	t	Sig.
(Constant)	.308	.297	1.038	.300
Dog Bond	-.002	.029	-.077	.938
Guilt- time/attention	.076	.012	6.424	<.001
Guilt factor – work	.034	.013	2.651	.008
Guilt factor – leave alone	.097	.016	6.072	<.001
Guilt factor – physical health	.034	.014	2.383	.018
Guilt factor – furniture	-.010	.012	-.844	.399
Gender	.205	.081	2.536	.012
Discrepancy ideal vs actual	.352	.097	3.626	<.001
WFC-D	.253	.037	6.789	<.001

Table S2. Reported Compensatory Behaviors, Feelings, and Thoughts as a Result of Dog-Related Guilt

	Never/rarely	Sometimes	A fair amount	A great deal/always
Say no to evening social events because you feel guilty leaving your dog at home (n=559)	326 (58%)	156 (28%)	59 (9%)	27 (5%)
Say no to weekend social events because you feel guilty leaving your dog at home (n=562)	316 (56%)	155 (28%)	51 (9%)	40 (7%)
Say no to vacations because you feel guilty leaving your dog at home (n=523)	272 (52%)	122 (23%)	67 (13%)	62 (12%)
Bring your dog to events and/or work because you feel guilty leaving your dog at home	304 (62%)	118 (24%)	47 (10%)	22 (5%)
Spend time with your dog at the expense of other family members because you feel guilty (n=541)	355 (66%)	127 (24%)	42 (8%)	17 (3%)
Remind yourself that your dog has a good life when you feel guilty (n=565)	136 (24%)	152 (27%)	149 (26%)	128 (23%)
Feel resigned to feeling guilty about your dog (n=551)	318 (58%)	155 (28%)	51 (9%)	27 (5%)

Table S3. Kruskal-Wallis Test Results Assessing the Association between GAPS-D Scores and Compensatory Behaviors, Feelings and Thoughts

	H (df)	P
Say no to evening social events because you feel guilty leaving your dog at home (n=559)	75.40 (3)	<0.001
Say no to weekend social events because you feel guilty leaving your dog at home (n=562)	66.88 (3)	<0.001
Say no to vacations because you feel guilty leaving your dog at home (n=523)	65.33 (3)	<0.001
Bring your dog to events and/or work because you feel guilty leaving your dog at home	42.38 (3)	<0.001
Spend time with your dog at the expense of other family members because you feel guilty (n=541)	78.98 (3)	<0.001
Remind yourself that your dog has a good life when you feel guilty (n=565)	55.16 (3)	<0.001
Feel resigned to feeling guilty about your dog (n=551)	70.31 (3)	<0.001
